# Evaluation of a Rapid Anisotropic Model for ECG Simulation

**DOI:** 10.3389/fphys.2017.00265

**Published:** 2017-05-02

**Authors:** Simone Pezzuto, Peter Kal'avský, Mark Potse, Frits W. Prinzen, Angelo Auricchio, Rolf Krause

**Affiliations:** ^1^Center for Computational Medicine in CardiologyLugano, Switzerland; ^2^Faculty of Informatics, Institute of Computational Science, Università della Svizzera ItalianaLugano, Switzerland; ^3^Department of Biomeasurements, Institute of Measurement Science, Slovak Academy of SciencesBratislava, Slovakia; ^4^Electrophysiology and Heart Modeling Institute IHU LIRYCPessac, France; ^5^Inria Bordeaux Sud-OuestTalence, France; ^6^Department of Physiology, Cardiovascular Research Institute Maastricht, Maastricht UniversityMaastricht, Netherlands; ^7^Division of Cardiology, Fondazione Cardiocentro TicinoLugano, Switzerland

**Keywords:** ECG, eikonal model, lead fields, bidomain modeling, patient-specific modeling, electrophysiology

## Abstract

State-of-the-art cardiac electrophysiology models that are able to deliver physiologically motivated activation maps and electrocardiograms (ECGs) can only be solved on high-performance computing architectures. This makes it nearly impossible to adopt such models in clinical practice. ECG imaging tools typically rely on simplified models, but these neglect the anisotropic electric conductivity of the tissue in the forward problem. Moreover, their results are often confined to the heart-torso interface. We propose a forward model that fully accounts for the anisotropic tissue conductivity and produces the standard 12-lead ECG in a few seconds. The activation sequence is approximated with an eikonal model in the 3d myocardium, while the ECG is computed with the lead-field approach. Both solvers were implemented on graphics processing units and massively parallelized. We studied the numerical convergence and scalability of the approach. We also compared the method to the bidomain model in terms of ECGs and activation maps, using a simplified but physiologically motivated geometry and 6 patient-specific anatomies. The proposed methods provided a good approximation of activation maps and ECGs computed with a bidomain model, in only a few seconds. Both solvers scaled very well to high-end hardware. These methods are suitable for use in ECG imaging methods, and may soon become fast enough for use in interactive simulation tools.

## 1. Introduction

The cardiac muscle or myocardium consists of electrically active muscle cells (myocytes) connected to each other by gap junctions, embedded in extracellular fluid and other structures. The current state of the art in forward simulation of the electrocardiogram (ECG) is to use a bidomain reaction-diffusion model to simulate propagating electrical activation and repolarization as well as the potential field in the torso (Vigmond et al., 2007). The bidomain model (Henriquez, 2014) describes the electrical behavior of the cardiac tissue using two co-located domains to represent the interpenetrating networks of intracellular and extracellular space. The conductivity of each domain can be described by a conductivity tensor field **G**_*i*_ for the intracellular, and **G**_*e*_ for the extracellular domain, respectively. These tensors have different degrees of anisotropy (Roth, 1997). The two domains are separated by the volume-averaged cell membrane, but electrically connected to one another through the ion channels in this membrane. The bidomain model can be described by a reaction-diffusion system

(1), (2), (3) $$\left\{ \begin{array}{l} \nabla \cdot (G_{\text{i}}\nabla (V_{\text{m}}+\phi_{\text{e}}))=\beta (C_{\text{m}}\frac{\partial V_{\text{m}}}{\partial t}+I_{\text{ion}}(V_{\text{m}},w))-I_{\text{stim}},\\ \nabla \cdot ((G_{\text{i}}+G_{\text{e}})\nabla \phi_{\text{e}})=-\nabla \cdot (G_{\text{i}}\nabla V_{\text{m}}),\\ \frac{\partial w}{\partial t}=F(V_{\text{m}},w), \end{array}\right.$$

where *V*_m_ is the transmembrane potential, ϕ_*e*_ the potential in the extracellular domain, β the surface-to-volume ratio of the cell membrane, *C*_m_ the membrane capacitance per unit area, *I*_ion_ a non-linear function representing the sum of all transmembrane ionic currents, **w** a vector of state variables for the ionic currents, **F** a non-linear function, and *I*_stim_ an applied stimulus current. Equation (1) describes the current flow across the membrane and can be used together with Equation (3) to integrate the *V*_m_ distribution. Equation (2) describes the conservation of charge and can be used to solve ϕ_*e*_ from a given *V*_m_.

The functions *I*_ion_ and **F** represent the so-called membrane model. If a physiologically detailed membrane model is chosen and the torso model is sufficiently detailed, this allows highly realistic ECGs to be simulated.

Much simpler models are presently being used inside inverse models, which aim at recovering cardiac events from non-invasive signals (Ramanathan et al., 2004; van Dam et al., 2009; Tysler and Svehlikova, 2013; Wang et al., 2013; Erem et al., 2014), and in interactive ECG-simulation tools (van Oosterom et al., 2011). Typically, these models assume predefined propagation velocities and predefined action potential waveforms. They account for anisotropic propagation but neglect the effects of anisotropic myocardial conductivity on the relation between activation and the ECG. Whereas a bidomain reaction-diffusion solution for a single heart beat takes in the order of a million core-seconds and is typically run on high-performance computing (HPC) resources, simplified methods can obtain a solution in less than a second on a single CPU core. Our purpose is to propose an ECG simulation method that is intermediate between these two extremes.

The conductivities play three roles in the ECG:

1. They determine the conduction velocity, which will also be anisotropic;
2. They determine the current dipole density resulting from a voltage gradient; and
3. They determine how the resulting current flows through the heart and torso and thus what voltages will be measured by the ECG electrodes.

While virtually all models nowadays account for anisotropic propagation, rapid ECG models typically neglect the second and third role of the anisotropic conductivities. The major reason for this is that the conductivities are non-uniform: the fiber orientation rotates around the cavities of the heart and also changes transmurally. To account for it, therefore, a volumetric discretization of the heart and torso is necessary. By assuming isotropy, in contrast, existing methods simplify the problem to one on the surface of the muscle (Geselowitz, 1992). However, the anisotropic conductivity has profound effects on the ECG, especially on the standard precordial electrodes, which are located near the heart (Potse et al., 2009) and which are appreciated by cardiologists for the detailed information they provide. We hypothesized that with today's computer technology one can build a rapid, fully anisotropic ECG model that is fast enough to be employed inside an inverse method.

One possible way to reduce the computational intensity of the bidomain model is to use an eikonal model (Colli Franzone and Guerri, 1993; Pullan et al., 2002) for the evolution of the excitation wavefront. The excitation wavefront represents a thin depolarized region of cardiac cells and an eikonal equation can be used to compute the activation time at which a wavefront reaches a given point in the myocardium. The eikonal equation belongs to a broad class of Hamilton-Jacobi equations (Bornemann and Rasch, 2006) and can be formally derived by a perturbation argument applied to the bidomain equations (Colli Franzone and Guerri, 1993). Therefore, both approaches can be compared (Pullan et al., 2002).

The method we propose here uses a volumetric anisotropic eikonal propagation model, pre-computed action potentials, and an ECG simulation based on dipole sources and a set of transfer functions known historically as lead fields. The volumetric eikonal model, in contrast to the surface models used by others (van Dam et al., 2009), provides us with an activation sequence throughout the cardiac volume, which we need to compute current dipoles at different locations with different fiber orientations. The numerical scheme adopted is guaranteed to converge to the correct viscosity solution of the eikonal equation (Bornemann and Rasch, 2006), which in turn correctly defines the fastest path between points through its gradient.

Precomputed action potentials are assigned and shifted according to the computed activation times, resulting in a field of transmembrane potentials at any desired time instant. For each time instant, the current dipoles are computed with an anisotropic formula, and the ECG potentials are computed from them using a set of transfer coefficients. The transfer coefficients are computed once for each model by solving a bidomain problem in the full torso, again taking all anisotropic conductivities into account. This method of ECG simulation is mathematically equivalent with full bidomain solutions of the torso potential based on monopole or dipole sources (Potse et al., 2009; Jacquemet, 2015). The only simplification of our model with respect to a full reaction-diffusion solution is the assumption of fixed but possibly heterogeneous action potential waveforms and conduction velocities that is inherent in the eikonal formulation.

The eikonal model allows for the use of a much coarser spatial resolution of 1 mm (Colli Franzone and Guerri, 1993) than the 0.1 mm scale required to reliably reconstruct the sharp upstroke of the action potential by a reaction-diffusion model (Clayton et al., 2011; Pezzuto et al., 2016). As a result much shorter computation times can be reached. The spatial discretization at a resolution of 1 mm is high enough to resolve the local fiber orientation and allow an anisotropic computation of the dipole sources. Thus, the method is able to account for the influence of anisotropic conductivity on the ECG, as well as on the propagation velocity.

The method was entirely implemented in C, C++ and CUDA so it could run efficiently on general-purpose graphics processing units (GPGPU).

Using a reaction-diffusion and bidomain model as a reference, we evaluated the proposed model in terms of the accuracy of the simulated activation sequence and ECG.

Evaluations were performed using six patient-specific heart-torso models. Finally, we evaluated the parallel scalability of the methods on GPGPUs.

## 2. Materials and methods

### 2.1. Reference model

A “monodomain” reaction-diffusion model of the heart coupled to a bidomain heart-torso model was used as reference. The monodomain model can be derived from the bidomain model by assuming that the two domains have the same anisotropy ratio, **G**_e_ = λ**G**_i_, with λ a constant scalar. Then Equations (1) and (2) reduce to a single reaction-diffusion equation

(4) $$\nabla \cdot ({\text{G}}_{\text{m}}\nabla V_{\text{m}})=\beta \left(C_{\text{m}}\frac{\partial V_{\text{m}}}{\partial t}+I_{\text{ion}}\right)-I_{\text{stim}}$$

where **G**_m_ is an effective conductivity. Under the equal anisotropy assumption, ${\text{G}}_{\text{m}}=\frac{\lambda }{1+\lambda }\;{\text{G}}_{\text{i}\cdot }$ A monodomain reaction-diffusion model is a very good approximation for its bidomain counterpart even when the equal anisotropy assumption does not hold (Potse et al., 2006; Nielsen et al., 2007; Bishop and Plank, 2011; Coudière et al., 2014), provided that the effective conductivity is chosen as ${\text{G}}_{\text{m}}={\text{G}}_{\text{e}}{\left({\text{G}}_{\text{i}}\;+\;{\text{G}}_{\text{e}}\right)}^{-1}{\text{G}}_{\text{i}}.$ Spatial discretization was performed with a semi-structured finite-difference mesh with 0.2 mm resolution. The time step was 0.01 ms for both the diffusion and the reaction, respectively solved with explicit Euler and Rush-Larsen schemes. The activation time was computed as the time when *V*_m_ became positive.

To compute the ECG, we solved the equation

(5) $$\nabla \cdot [({\text{G}}_{\text{i}}+{\text{G}}_{\text{e}})\nabla \phi_{\text{e}}]=-I_{\text{w}},$$

in a 3d finite-difference full torso model at 1-mm resolution, including a downsampled heart model (Potse et al., 2009). The term *I*_w_ is a projection of the transmembrane current ∇·(**G**_i_∇*V*_m_) onto the coarse mesh (Potse and Kuijpers, 2010). The surface ECGs were derived by evaluating the potential at the electrode locations.

All reference computations were performed with the propag-5 software (Potse et al., 2006; Krause et al., 2012) and ran on a Cray XC40 computer.

### 2.2. Eikonal model

To compute the activation sequence of the heart we used a non-linear first-order Hamilton-Jacobi eikonal equation in the form

(6) $$\{\begin{array}{ll} \frac{\alpha }{\sqrt{\beta }}\sqrt{{\text{G}}_{\text{m}}\nabla \Psi \cdot \nabla \Psi }=1, & x\in \Omega \setminus {\left\{s_{k}\right\}}_{k=1}^{K},\\ \Psi (s_{k})=\tau_{k} & k=1,\ldots ,K. \end{array}$$

where Ψ(*x*) is the activation time at the point *x* ∈ Ω and α = α(*x*) is a scaling parameter for the conduction velocity. Propagation is initiated at *K* > 0 early activation sites *s*_*k*_ with initial time τ_*k*_.

The Equation (6), which assigns a locally constant front velocity, is one of the simplest eikonal models. More sophisticated versions of the eikonal equation are also available, where the velocity of the front depends also on the curvature of the front itself and higher-order curvature terms, or where the propagation is defined by a Finsler metric rather than a Riemannian metric (Colli Franzone et al., 2014).

In general, the scaling parameter α is the velocity that the front would have if the conductivity and β were set to one. Indeed, given a propagation direction **p**, the conduction velocity arising from the reaction-diffusion model (Equation 4) is proportional to

(7) $$\frac{\sqrt{{\text{G}}_{\text{m}}p\cdot p}}{\sqrt{\beta }}$$

which is exactly the conduction velocity given by the eikonal model for α = 1. α depends only on the membrane capacitance and the ionic model, in particular on the sodium-channel conductivity and gating dynamics.

**Figure 1:**
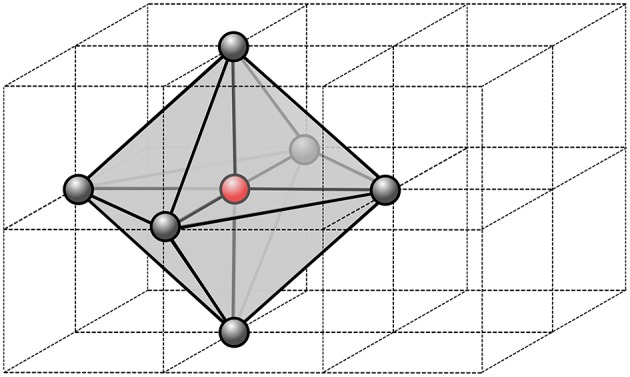
**Stencil of a single node of the computational grid**. In 3D, we use 6 neighbor nodes from which we define a virtual patch of tetrahedral element. Within each tetrahedron, the parameters are assumed constant, thus the fastest path is always a segment.

The implementation of our eikonal solver is based on a local variational principle proposed by Bornemann and Rasch (2006). Let δ(*y, x*) be the travel time between points *y* and *x*, computed with respect to a local metric, and $I_{\omega }$ a linear Lagrange interpolation operator restricted to a patch ω. The numerical solution $\Psi_{h}={\left\{\Psi_{h,i}\right\}}_{i=1}^{N}$, with Ψ_*h,i*_ the activation time at the node *x*_*i*_ of the mesh, is iteratively updated for each node by a local map Λ defined as follows:

(8) $$\Psi_{h,i}^{j+1}=\;\Lambda_{i}(\Psi_{h}^{j}):=\{\begin{array}{ll} \underset{y\in \partial \omega (x_{i})}{\min}\{\delta (y,x_{i})+I_{\omega (x_{i})}(\Psi_{h}^{j})(y)\} & \text{if }x_{i}\neq s_{k},\\ \tau_{k} & \text{if }x_{i}=s_{k}, \end{array}$$

for *i* = 1, …, *N*. The nodal value Ψ_*h,i*_ is updated using a patch ω(*x*_*i*_) of tetrahedral elements from the neighboring nodes (see Figure 1). The minimum in Equation (8) is taken over the polyhedral boundary ∂ω(*x*_*i*_) of the patch. The new value of Ψ_*h,i*_, if not enforced as boundary condition, is the minimum time at *x*_*i*_ traveling from the boundary of the patch.

The quantity δ(*y, z*) is defined as the travel time along the fastest path γ from *y* to *z* with respect to metric $\text{C}\left(x\right)=\beta \left(x\right)\alpha^{-2}\left(x\right){\text{G}}_{m}^{-1}\left(x\right)$:

(9) $$\delta (y,z):=\underset{\gamma \in C^{1}([0,1],{\mathbb{R}}^{3})}{\inf}\left\{\begin{array}{l} \int_{0}^{1}||\dot{\gamma }(t)|{|}_{C(\gamma (t))}\text{ d}t:\gamma (0)=y,\\ \;\gamma (1)=z \end{array}\right\}.$$

In order to compute it, we discretize the metric **C**^−1^(*x*) as piecewise-constant on the patch ω(*x*), i.e., its value is constant within each tetrahedron. Then the fastest path for each tetrahedron is the segment connecting *y* and *z*, and the travel time is ||*z* − *y*||_**C**_.

The minimization problem in Equation (8) is solved by taking the minimum of the minima for each triangular face t of ∂ω(*x*_*i*_):

(10) $$\underset{\text{t}\in \partial \omega (x_{i})}{\min}\underset{y\in \text{t}}{\min}\{||x_{i}-y|{|}_{C}+\;I_{f}(\Psi_{h}^{j})(y)\}.$$

The minimization over the single triangular face is a convex optimization problem, hence the minimum is unique.

It is possible to show that the map Λ has a unique fixed point (Bornemann and Rasch, 2006). Moreover, the fixed point converges to the correct viscosity solution of the eikonal equation as *h* → 0. The convergence rate is generally O(h) or less, due to the low regularity of the solution, which is only Lipschitz continuous even for a regular domain and coefficients.

### 2.3. ECG

Each ECG lead potential *V*(*t*) at time *t* was computed as

(11) $$V(t)=\int_{\Omega }\nabla Z(x)\cdot {\text{G}}_{\text{i}}(x)\nabla V_{\text{m}}(x,t)\text{ d}x$$

where Ω is the heart domain and *Z*(*x*) is the lead field (McFee and Johnston, 1953) of the specific ECG lead. The lead field is the potential field created by a unit current applied at the electrode locations (Geselowitz, 1989)

(12) $$\nabla \cdot (\text{G}\nabla Z)=\{\begin{array}{ll} -1 & \text{at the positive electrode},\\ \;1 & \text{at the negative electrode},\\ \;0 & \text{else where}, \end{array}$$

where **G** = **G**_i_ + **G**_e_ is the bulk conductivity (**G**_i_ = 0 outside the heart) (Potse et al., 2009). Equation (12) was solved for *Z* with propag-5 (Potse et al., 2006; Krause et al., 2012), using the complete heart-and-torso model at 1-mm resolution. Both the transfer function and the dipole sources (**G**_i_∇*V*_m_) were computed with fully anisotropic conductivity values.

To obtain *V*_m_(*x, t*) from Ψ(*x*) we used a fixed action potential waveform *U*(*t*):

(13) $$V_{\text{m}}(x,t)=U(t-\Psi (x)).$$

However, we may also introduce position-dependent action potentials U(t−Ψ(x),x). In the case of Equation (13), thanks to the co-area formula, the ECG reads as follows

(14) $$V(t)=\int_{-\infty }^{\infty }U^{\prime }(t-\xi )\left(-\int_{\Psi^{-1}(\xi )}\nabla Z\cdot {\text{G}}_{\text{i}}\text{n d}\Sigma \right)\text{d}\xi =(U^{\prime }*w)(t),$$

that is, the convolution of the first derivative of *U*(*t*) and the function *w*(*t*) defined as:

(15) $$w(t):=-\int_{\Psi^{-1}(\xi )}\nabla Z\cdot {\text{G}}_{\text{i}}\text{n d}\Sigma ,$$

where Ψ^−1^(*t*) = {*x* ∈ Ω: Ψ(*x*) = *t*} is the front surface within the heart and n is its normal oriented in the propagation direction. The function *w*(*t*) is exactly the ECG *V*(*t*) when the template action potential *U*(*t*) is the Heaviside function, representing a sharp interface between the depolarized and the resting tissue.

We implemented two methods for the computation of the ECGs: one based on the general formula (11), termed “simple method” (SM), and one based on formulas (14) and (15), named “fast method” (FM). The former can account for action potential heterogeneity, which is important for the T-wave in the ECG. When focusing on the QRS complex, the main differences between the proposed methods are the way the cardiac sources are computed and resulting execution speed of the computation of the ECGs.

The SM computes the ECG by direct evaluation of Equation (11). The heart domain Ω is discretized into voxels v ∈ Ω_*h*_. The functions *Z* and *V*_m_ are approximated by piecewise linear functions *Z*_*h*_ and (*V*_m_)_*h*_, respectively, thus ∇*Z*_*h*_ and ∇(*V*_m_)_*h*_(*t*) are piecewise constant vectors within each voxel v. The gradient of *V*_m_ is computed for each time step. The intracellular conductivity **G**_i_ is assumed piecewise-constant. The time window is prescribed and the time step τ > 0 is fixed (usually 1 ms). Consequently, the numerical approximation *V*_*n*_ of the ECG at time *t* = *nτ* reads:

(16) $$\begin{array}{l} V(n\tau )\approx V_{n}=\sum_{\text{v}\in \Omega_{h}}|\text{v}|\nabla Z_{h}{|}_{\text{v}}\cdot {\text{G}}_{\text{i}}{|}_{\text{v}}\nabla {\left(V_{\text{m}}\right)}_{h}(n\tau ){|}_{\text{v}},\\ \;n=0,\ldots ,N_{\tau }. \end{array}$$

The convergence rate with respect to *h* is quadratic, for smooth *Z*(*x*) and *V*_m_(*x, t*). In practice, *Z*_*h*_(*x*) is the solution of a discrete Laplace equation, which converges quadratically in the *L*^2^ norm to the analytical solution (far from the electrodes, where the solution is not singular). The action potential depends on the activation time Ψ_*h*_(*x*) computed with the eikonal equation, and the convergence rate is linear or less in the *L*^∞^ norm. Hence, the overall error of Equation (16) is dominated by the error on the activation time.

The FM, based on Equation (14), consists of two steps. First, the piecewise linear interpolants in time of *w*(*t*), *w*_*n*_ ≈ *w*(*nτ*), and of *U*(*t*), *U*_*n*_ ≈ *U*(*nτ*), are computed. Then, the surface over which the integral (Equation 15) is defined, is approximated with a triangular surface. This latter is produced from the activation map by means of the marching cubes algorithm (Lorensen and Cline, 1987; Newman and Yi, 2006), a well-known and commonly used algorithm for extracting triangulated isosurfaces from 3D scalar fields. It has the important advantage of being intrinsically parallel.

The interpolated function *w*_*n,h*_ reads as follows:

(17) $$w(n\tau )\approx w_{n,h}=-\sum_{\text{v}\in \Omega_{h}}(\nabla Z_{h}{|}_{\text{v}}\cdot {\text{G}}_{\text{i}}(\sum_{\text{t}\in T_{h}(\text{v},n)}|\text{t}|{\text{n}}_{\text{t}})),$$

where $T_{h}\left(\text{v},n\right)$ is the triangulation of the surface v∩Ψ^−1^(*nτ*), and n_t_ is the normal to the triangle t.

It is important to note at this stage that Ψ^−1^(τ*n*) ∩ v is empty for most of the values of *n*. Actually, it is non-empty for min_v_ Ψ_*h*_ ≤ τn ≤ max_v_ Ψ_*h*_. This helps to reduce the computational cost significantly. For instance, with τ = 1 ms, the activation front is found in the voxel on average only for 2 to 3 time instants. Moreover, the expression (Equation 17) can be further simplified since the normal n_t_ and the area |t| are related: let v_1,t_, v_2,t_ and v_3,t_ be the vertices of the triangle t, then we have

(18) $$|\text{t}|{\text{n}}_{\text{t}}=\frac{1}{2}({\text{v}}_{2,\text{t}}-{\text{v}}_{1,\text{t}})\wedge ({\text{v}}_{3,\text{t}}-{\text{v}}_{1,\text{t}}).$$

Finally, the second step is the convolution, approximated by

(19) $$V(n\tau )\approx V_{n}=\frac{1}{2}\sum_{p\in \mathbb{Z}}(U_{p+1}-U_{p-1})w_{n-p},$$

which results from a central finite difference approximation of *U*′(*t*) combined with the midpoint quadrature rule.

### 2.4. GPGPU implementation

The proposed computational methods for activation times and ECG were designed to run on GPGPU architectures. The power of GPGPU computation lies in a massive parallelism that can hide long-latency operations such as memory accesses. The latency hiding is possible thanks to the warp schedulers, which continuously switch among warps whose instructions are ready for execution.

The solvers were implemented in C and C++ with CUDA extension (Nickolls et al., 2008; Cheng et al., 2014), version 8.0. In all our experiments a single precision floating point representation of numbers was used, sufficient for stability and accuracy of the solvers.

The eikonal Equation (6) is solved by means of the Fast Iterative Method (FIM), proposed by Jeong and Whitaker (2008). Although a popular alternative for solving the eikonal equation is the Fast Marching Method (FMM) (Sethian, 1996), we opted for the FIM for two reasons: first, to increase the parallelization of the code and to make it suitable for GPGPU. Second, the updating of map (Equation 8) requires some care for anisotropic media in the FMM (Mirebeau, 2014).

Briefly, the FIM algorithm proceeds as follows: first, the activation map is initialized to +∞ except at the boundary points; then, we iterate until a list of *active nodes* is empty. At each iteration, threads operate on single active nodes of the grid. For each node, the local minimization problem (Equation 10) is solved, and the new value is assigned to the node, according to Equation (8). When all nodes have been updated, all the neighbors of the active nodes that are not converged are appended to the active list, and activation time is re-computed. Active nodes whose activation times do not differ from their previous values are removed from the active list and marked as converged.

The implementation of ECG computation with the SM and FM is similar. Each cube is assigned to an individual thread, and each thread updates the local contribution of one cube to the ECG. For the SM, the kernel iterates over time and computes *V*_*n*_ according to Equation (16). The time-independent term **G**_i_∇*Z* is evaluated only once by each thread.

**Figure 2:**
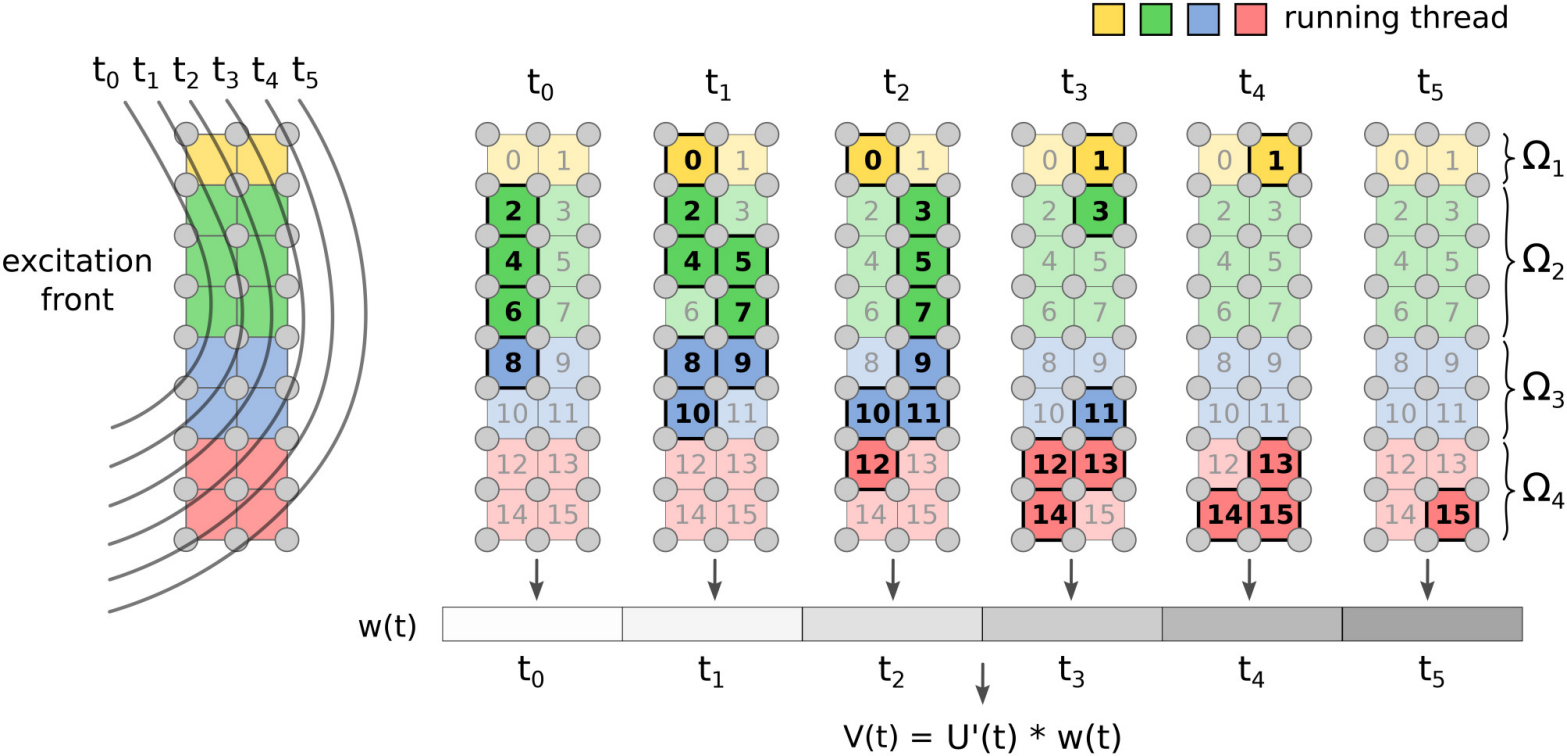
**Implementation of FM for ECG computation**. Each voxel of the grid updates the value of *w*(*t*) only for the time instants when the front is crossing it. Once all the voxels are done, the convolution is computed.

The FM has two kernels: one for the computation of *w*(*t*) Equation from Equation (15), and another for the convolution (Equation 14). The first kernel implements the marching cubes algorithm through a look-up table that stores all the possible cases on how the front intersects the voxel. The marching cubes look-up table and the template action potential are stored in texture memory for fast indexing. The array *w*_*n*_ is updated concurrently by the threads. Each voxel contributes to *w*_*n*_ only when the front is within it (see Figure 2).

The computational complexity in time of the SM is $O\left(L/h^{3}\cdot T_{max}/\tau \right)$, where *L* is the characteristic length of the activation wavefront and *T*_max_ is the maximum activation time. In the FM, the voxel contribution is evaluated roughly $\approx \frac{h}{\tau \theta }$ times, where θ is the average conduction velocity within the voxel. Thus, the complexity in this case is $O\left(L\theta^{-1}h^{-2}\tau^{-1}\right)$. The ratio between SM and FM complexity is ≈ *T*_max_θ/*h*.

Since the computations of individual ECG leads are independent from each other, we also utilized parallel CUDA streams to support simultaneous execution of multiple kernels computing multiple ECG leads. All memory transfers between the CPU and GPU are asynchronous, in order to support also overlapping of the data transfers with the kernel executions.

### 2.5. Model geometries

Patient-specific heart and torso geometries were created as described previously (Nguyên et al., 2015). Model parameters were tuned to each patient to match both the activation sequence on the endocardium and the twelve-lead ECG (Potse et al., 2014).

The model geometry was described in terms of a set of Catmull-Clark subdivision surfaces. The simulations were performed on semi-structured hexahedral meshes, i.e., while the basis of the mesh was regular, we did not store information for mesh nodes that did not play a role in the computation. These meshes were created by overlying the mesh base on the set of surfaces and assigning the properties of the mesh elements based on the surfaces in which they were included. Passive tissue characteristics were properties of elements, while active parameters and variables applied to nodes. The node properties followed from the element properties using a set of rules that ensured model consistency (Potse et al., 2006).

## 3. Results

### 3.1. Numerical assessment

In order to check the convergence of the method, we performed two numerical experiments. In both of them, we solved the eikonal equation on a tissue slab of size 1.5 × 2 × 2 cm, with a single pacing site at x_0_ = (0, 1, 1) cm.

In the first experiment, parameters were selected such that an analytical solution for both the activation times and the function *w*(*t*) defined in Equation (15) was available, and such that the ECG was easy to approximate with high accuracy. In particular, we considered fibers oriented along the *z*-axis. The ECG was computed with the lead-field function *Z*(*x, y, z*) = −*x* and an action potential defined as follows:

(20) $$U(t)={\text{V}}_{\text{ref}}+\frac{{\text{V}}_{\text{dep}}-{\text{V}}_{\text{ref}}}{2}(\tanh(2\frac{t}{\varepsilon_{\text{dep}}})-\tanh(2\frac{t-\text{APd}}{\varepsilon_{\text{rep}}})),$$

where the definitions and numerical values of the parameters are given in Table 1.

**Table 1:** **Values of the parameters adopted in the two convergence tests**.

**Parameter**	**Description**	**Value**
α	Conduction velocity scaling	2.0 cm ms^−1^ mS^−1/2^
β	Surface-to-volume ratio	1000 cm^−1^
σ_el_	Longitudinal extra-cellular conductivity	3 mS cm^−1^
σ_et_	Transverse extra-cellular conductivity	1.2 mS cm^−1^
σ_ec_	Cross extra-cellular conductivity	1.2 mS cm^−1^
σ_il_	Longitudinal intra-cellular conductivity	3 mS cm^−1^
σ_it_	Transverse intra-cellular conductivity	0.3 mS cm^−1^
σ_ic_	Cross intra-cellular conductivity	0.3 mS cm^−1^
V_ref_	Resting potential	−80 mV
V_dep_	Depolarization potential	30 mV
ε_dep_	Depolarization time-scale	1 ms
ε_rep_	Repolarization time-scale	100 ms
APd	Action potential duration	200 ms

The exact solution for the activation times reads:

(21) $$\Psi (\text{x})=\frac{1}{\alpha }\sqrt{\beta {\text{G}}_{\text{m}}^{-1}(\text{x}-{\text{x}}_{0})\cdot (\text{x}-{\text{x}}_{0})}.$$

The ECG solution is the convolution between the first derivative of the action potential (20) and the function *w*(*t*). The latter was computed exactly, being proportional to the difference in depolarized area between the two faces of the slab orthogonal to the *x*-axis:

(22) $$\begin{array}{l} w(t)\sim \mathrm{Area}(\{(y,z)\in {\left[0,2\right]}^{2}:\Psi (0,y,z)\leq t\})\\ \;-\mathrm{Area}(\{(y,z)\in {\left[0,2\right]}^{2}:\Psi (1.5,y,z)\leq t\}). \end{array}$$

The activated region on these two faces of the slab was always the intersection between an axis-aligned ellipse and a square. The corresponding area was derived from elementary geometry. See the Appendix in Supplementary Materials for further details and the explicit formula. The convolution was eventually computed numerically with high accuracy.

**Figure 3:**
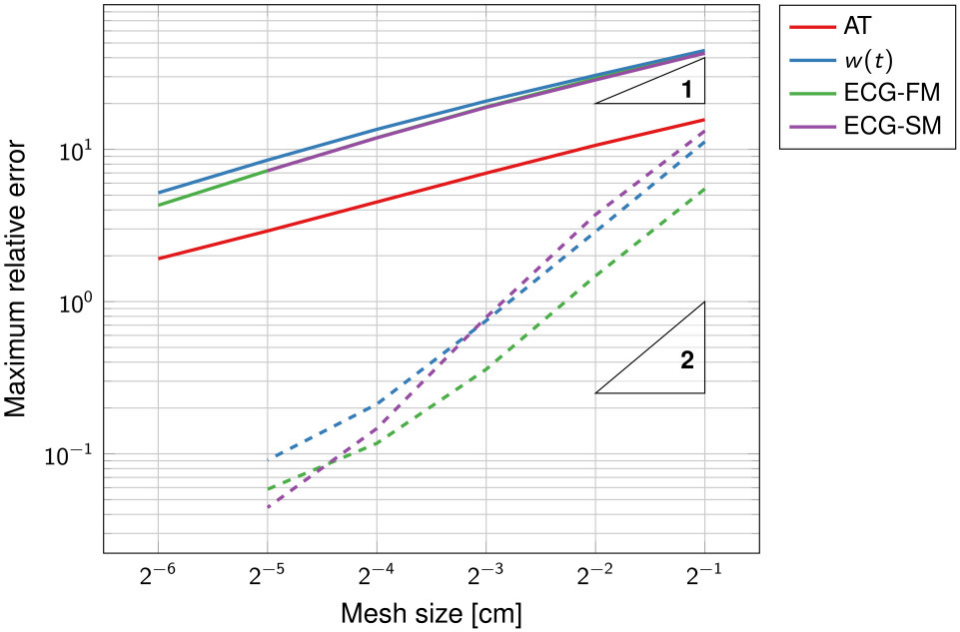
**Double-logarithmic plot of the error on the activation map, the function ***w***(***t***) and the ECG**. The dashed lines refer to the computation of the ECG using the exact activation map. The triangles are such that the slope of their longest side is equal to the indicated number, in logarithmic coordinates. The convergence rate of the solid lines is sub-linear.

The convergence rate for the activation map was $O\left(h^{0.6}\right)$, as reported in Figure 3. This affected the ECG computation as well. This sub-linear convergence can be explained by the low regularity of the solution, which is singular at the source point. We also tested the ECG computation with the exact solution (Equation 21), in order to assess the convergence rate of the SM and FM alone. In this case, we observed quadratic convergence rate, as expected from the theory.

The SM and the FM perform similarly, with FM being slightly more accurate than SM at coarser grids. The high accuracy of the SM is surprising because the template action potentials had very steep gradients in space (0.2 mm thick), clearly not captured by the coarse grid. But a closer inspection of the method showed that while the current per unit volume was not correctly approximated, the total current once integrated over the voxel was accurate.

**Figure 4:**
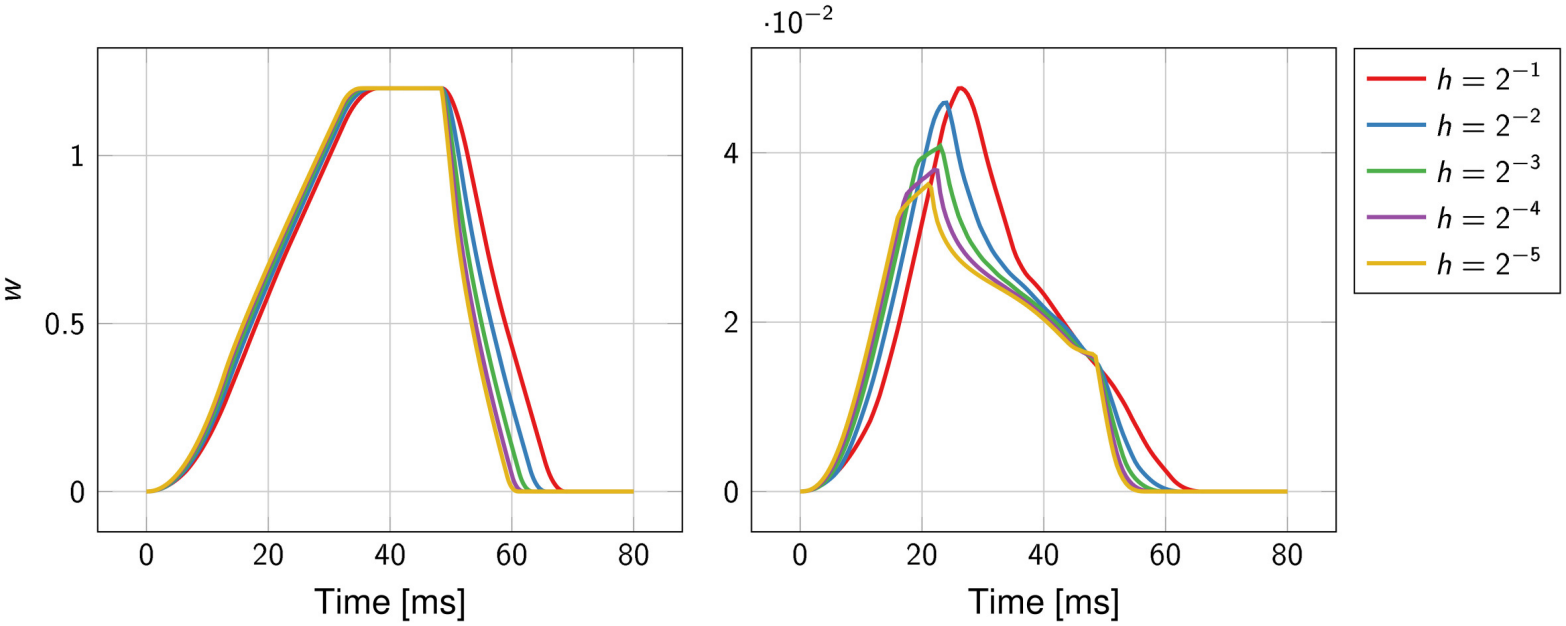
**Convergence history of the function ***w***(***t***) for the first experiment (on the left)** and the second experiment **(on the right)**. The mesh size *h* ranges from 2^−1^ cm to 2^−5^ cm.

The function *w*(*t*), defined in Equation (15), was well-approximated already at the coarsest grid (Figure 4). As the mesh was refined, the support of the function narrowed, reflecting the fact that the total activation time was shortening.

The second numerical experiment mimicked a transmural specimen of left ventricle. Fibers were parallel to the *yz*-plane and formed an angle with respect to the *y*-axis linearly varying from −π/3 at *x* = 0 to π/3 at *x* = 1.5. Parameters were as in the previous experiment. The lead field function was the solution of the problem (Equation 12) in ℝ^3^ with uniform conductivity σ_t_ = 2 mS cm^−1^ and reads as follows:

(23) $$Z(\text{x})=\frac{1}{4\pi \sigma_{\text{t}}}(\frac{1}{\left|\text{x}-{\text{x}}^{-}\right|}-\frac{1}{\left|\text{x}-{\text{x}}^{+}\right|}),$$

with negative terminal x^−^ = (3, 1, 1) cm and positive terminal x^+^ = (−1, 1, 1) cm.

In contrast to the first experiment, an analytical solution for the activation times, and thus for the ECG, was not available. The error was estimated using a solution obtained at a resolution of 2^−8^ ≈ 0.004 cm as reference. The convergence rate was roughly $O\left(h^{0.9}\right)$; higher than in the previous experiment but still sub-linear. The function *w*(*t*) and the ECG were not correctly captured at the coarser resolution, as certified by Figure 4. This was likely due to the too coarse representation of the fiber field.

From these experiments we concluded that a resolution of 1 or 0.5 mm for the eikonal solver provides reasonably accurate solutions, with a relative error lower than 5%.

### 3.2. Comparison to bidomain with a tissue slab

The quantitative evaluation of the modeling error introduced by the proposed model with respect to the reference reaction-diffusion (R-D) model was conducted on a simple geometry but with physiologically motivated heterogeneities in the parameters. For this comparison we focused on the depolarization phase.

**Figure 5:**
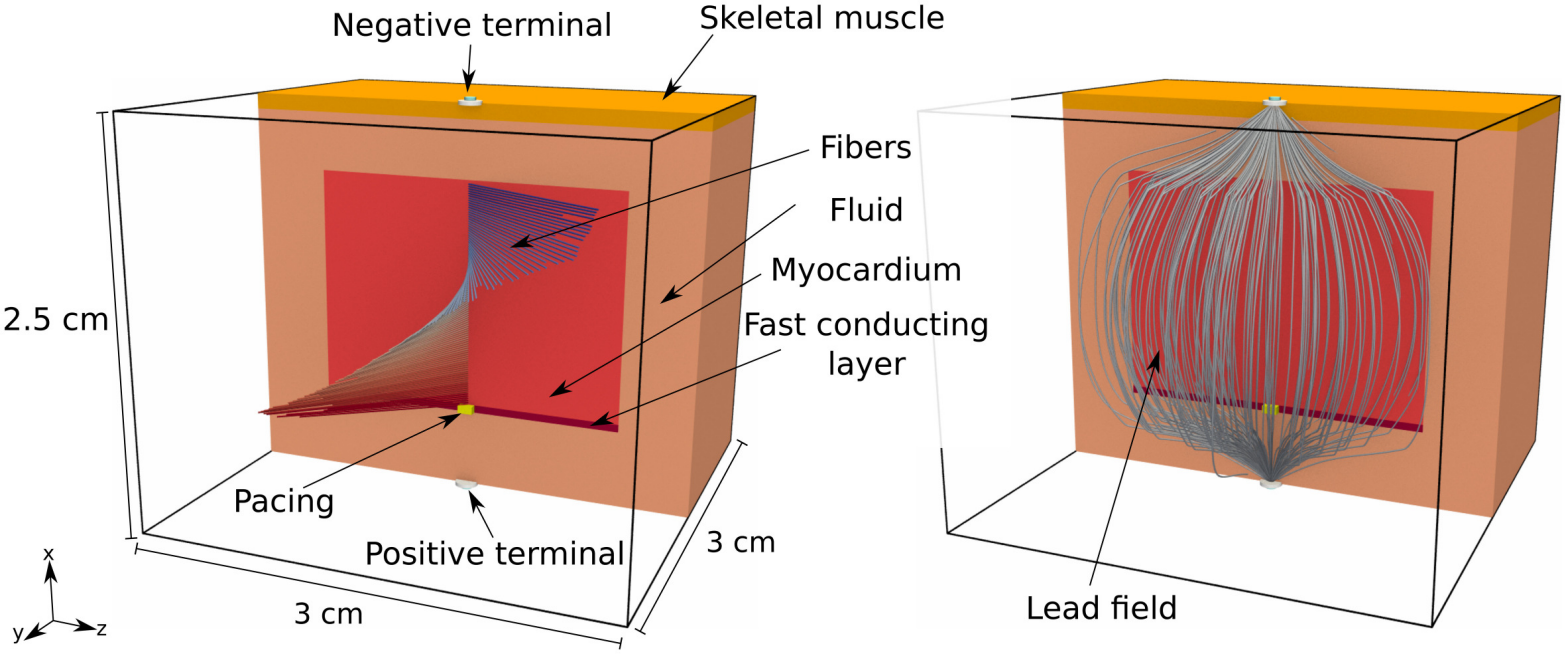
**Pictorial representation of the geometry of the tissue slab, embedded in a heterogeneous conductive bath, and the corresponding lead field**. The tissue slab is 1.5 × 2 × 2 cm and positioned at the center of the bath. At the bottom of the slab there is a rapidly conducting layer, 0.5 mm thick, where the surface-to-volume ratio is reduced. At the top of the bath there is a 1 mm thick layer of skeletal muscle, with a reduced electric conductivity with respect to the underlying conductive medium (labeled “fluid” in the picture). There is a single early activation site at the bottom of the slab.

We considered a tissue slab shaped as in the previous section and embedded at the center of an electrically conductive bath of dimension 2.5 × 3 × 3 cm. The fiber organization in the slab was exactly as in the second experiment above, but we also introduced a 0.5 mm thick rapidly conducting layer at the bottom face of the slab. The propagation was initiated in a 0.5 × 1 × 1 mm volume at the center of the rapidly conducting layer. The bath was composed of two homogeneous and isotropic media, one 1 mm thick placed on top of the bath, which we named “skeletal muscle,” and the other, called “fluid,” filling the remaining part of the volume. The activation time was measured only in the tissue slab. A bipolar ECG was also produced by taking the potential difference between two electrodes placed at the centers of the top and bottom faces of the bath. An overview of the experimental setting is depicted in Figure 5.

The parameters in the R-D model and the proposed model, summarized in Table 2, were the same when applicable. The electric conductivity in the tissue slab and the fast conducting layer was transversely isotropic, while it was assumed fully isotropic but heterogeneous in the bath. The surface-to-volume ratio was reduced in the fast layer in order to increase the conduction velocity with a factor 2. The ionic model, only present in the R-D model, was TNNP (ten Tusscher et al., 2004).

**Table 2:** **Electric conductivity (mS cm^**−1**^) and surface-to-volume ratio (cm^**−1**^) of the media employed in the comparison test**.

**Medium**	**σ_il_**	**σ_it_**	**σ_el_**	**σ_et_**	**β**
Tissue	3.0	0.3	3.0	1.2	800
Fast	3.0	0.3	3.0	1.2	356
Muscle	–	–	0.44	0.44	–
Fluid	–	–	6.0	6.0	–

The value for α in the eikonal model was obtained from the conduction velocity θ observed in a 1d preparation of the R-D model (Equation 4) through the relation

(24) $$\alpha =\sqrt{\frac{\beta }{\sigma }}\cdot \theta .$$

For instance, with β = 1,000 cm^−1^, σ = 1.5 mS/cm we observed θ = 0.075935 cm/ms, the latter being 4-digit accurate, hence α = 1.961 cm ms^−1^ mS^−1/2^. From the same 1d preparation we also extracted the template action potential *U*(*t*) by evaluating the resulting transmembrane potential.

The propagation was triggered differently in the two models. In the eikonal model, we assumed that the pacing region was activated with an initial time of 1.12 ms, the latter obtained from the monodomain simulation. This delay is associated to the time required by the current injection to start the propagation.

In order to minimize the numerical error with respect to the modeling error, we performed the comparison on a uniform grid with voxels of side 0.05 mm. The eikonal model and the monodomain equation were solved only in the tissue slab. The forward bidomain problem was eventually solved including also the bath, and injecting the transmembrane currents in the tissue slab. The lead field, depicted in the right panel of Figure 5, was computed from Equation (12) with a second order finite-difference scheme on a 0.1 mm spaced uniform grid and then interpolated at 0.05 mm resolution.

**Figure 6:**
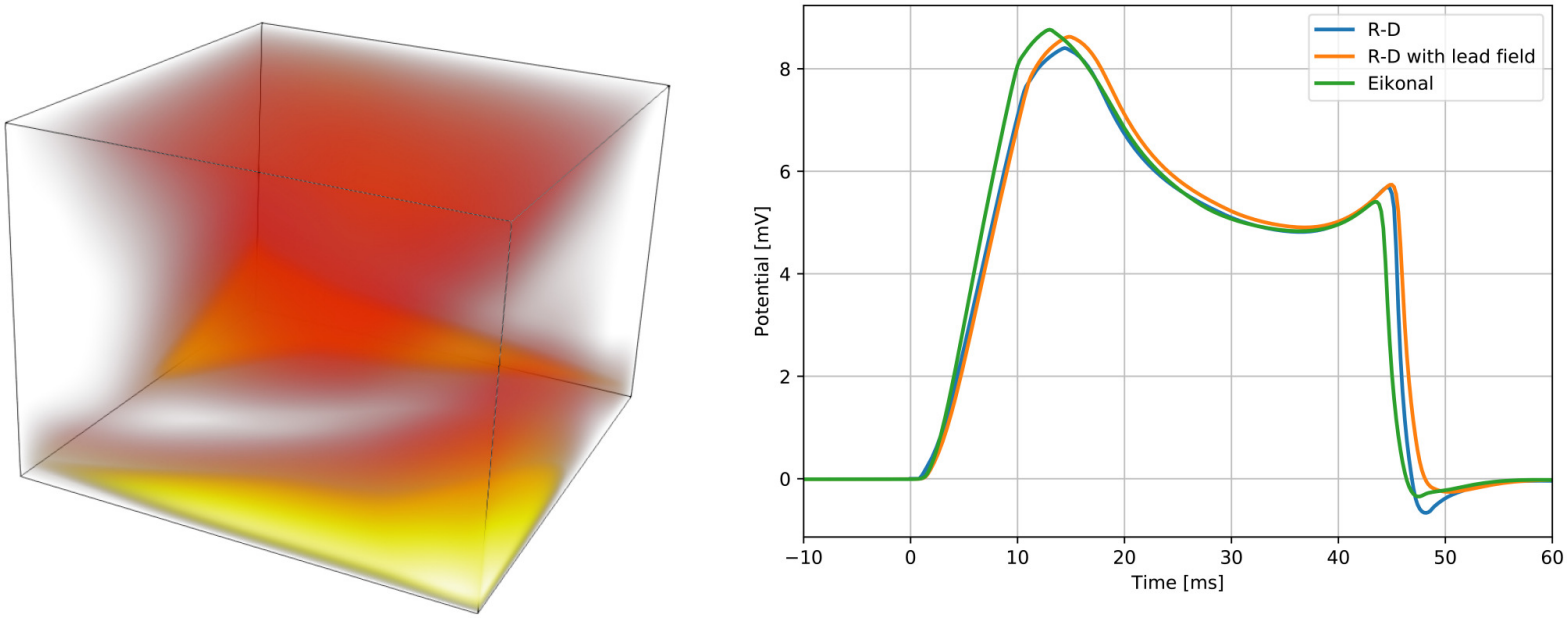
**Spatial modeling error between the proposed model and the reaction-diffusion model (left panel)**, and QRS complexes of the simulated ECGs **(right panel)**. On the left panel, the error is presented with a “heat map,” being brighter where the error is larger. The brightest area of the map corresponds to an error of 3 ms. On the right panel, we report the QRS complex simulated by the reaction-diffusion model, by the proposed model, and by an intermediate approach that consists in using the lead field approximation with the activation map produced by the monodomain equation.

The results are reported in Figure 6. In the R-D model, the slab fully depolarized after 50.04 ms, while in our model the latest activation was at 49.48 ms. The maximum absolute and the L^2^ errors were both around 3 ms, and for 95 % of the nodes the error was lower than 2 ms. In the R-D model, the activation occurred later than the activation from the eikonal model for all the nodes. Moreover, the largest error was localized at the boundaries and specifically in the fast layer.

We computed three ECGs in total: one with the R-D model, one with the proposed model, and one using the lead-field approach with the activation map obtained from the R-D model. The third was computed to evaluate the modeling error introduced by assuming a template action potential. The ECGs were very similar in shape and amplitude. In the terminal part of the QRS complex from the R-D model we observed a deeper inflection than in the other cases, and this seemed to be due to the assumption (Equation 13), since this was absent also in the case of the R-D model with lead field.

Finally, we performed the same comparison for a coarser grid resolution, observing a longer QRS duration in the R-D model. This is consistent with the fact that the finite-difference scheme underestimates the conduction velocity at coarser resolution (Pezzuto et al., 2016). Differences in the activation map for the eikonal model at coarser resolution were negligible.

### 3.3. Comparison to bidomain with patient-tailored anatomy

Similarly to the previous section, we conducted an extensive comparison between the proposed model and the reference model on six patient-tailored anatomies, assuming the same parameters for both the models. Patients included in this study had a clinical indication for CRT; the ECG, cardiac resonance imaging, and an electrophysiological study, including 3D electro-anatomical mapping were clinically indicated. They were performed as part of the work-up for device selection, i.e., to evaluate scar location and extent, inducibility of ventricular tachycardia, and pacing site selection. The study was performed in compliance with the Declaration of Helsinki. The institutional review board approved the study protocol, and all patients gave oral and written informed consent for each investigation.

Characteristics of the heart meshes are listed in Table 3. In our heart models we assumed a layered structure of substances. Each ventricular model consisted of eight layers. Four transmural layers called *fast, endo, mid*, and *epi* were created in the right ventricle (RV) and another four similar transmural layers were created in the left ventricle (LV). During the experiments we set the conduction velocity to be faster in the *fast* layers of both ventricles, in order to mimic the presence of the Purkinje network, which our models did not include. Both in the eikonal model and in the reaction-diffusion model the increase in velocity was achieved by reducing the local value of β. For patient 3 only the RV was given a *fast* layer. Additional details are given in Potse et al. (2014) for patient 1 and 2 and in Nguyên et al. (2015) for patient 3 and 4.

**Table 3:** **Specification of the heart meshes for the eikonal model used in the experiments**.

**Patient**	**Mesh dimension**	**# Nodes**	**# Cubes**	**# EASs**
1	165 × 91 × 119	191,734	151,959	3
2	205 × 107 × 151	292,585	235,502	1
3	186 × 123 × 126	289,252	230,968	1
4	178 × 117 × 147	350,584	290,166	2
5	156 × 91 × 108	222,951	187,534	1
6	238 × 145 × 162	493,006	413,252	4

*Nodes are vertices in the mesh that represent active tissue. Cubes are non-void voxels of the geometry. The last column reports the total number of early activation sites (EASs) used in the experiments*.

The following electric conductivities were used for all the patients: torso 2 mS/cm; blood 6 mS/cm; lungs 0.5 mS/cm; skeletal muscle 3.55 mS/cm in the tangent plane and 0.44 mS/cm in the radial direction. The conductivities in the myocardium differed per patient as they were tailored to fit the measured ECG and activation sequence. A tuning was necessary for the activation time at the early activation sites (EASs) and the CV scaling parameter α, not present in the reaction-diffusion model. Left bundle branch block (LBBB) was assumed for all patients, and was modeled by placing the EASs only in the RV. The number of EASs for each patient is reported in Table 3.

The initiation of the activation in the R-D model was performed by injecting a transmembrane source current of 200 mA/cm^3^ for 2 ms on 1 mm^3^ of tissue centered at the corresponding EAS location. The currents can be applied at different times. In the eikonal solver, the activation time of the EASs was enforced as boundary condition and shifted in time in order to mimic the delay observed in the R-D model between current injection and depolarization.

**Figure 7:**
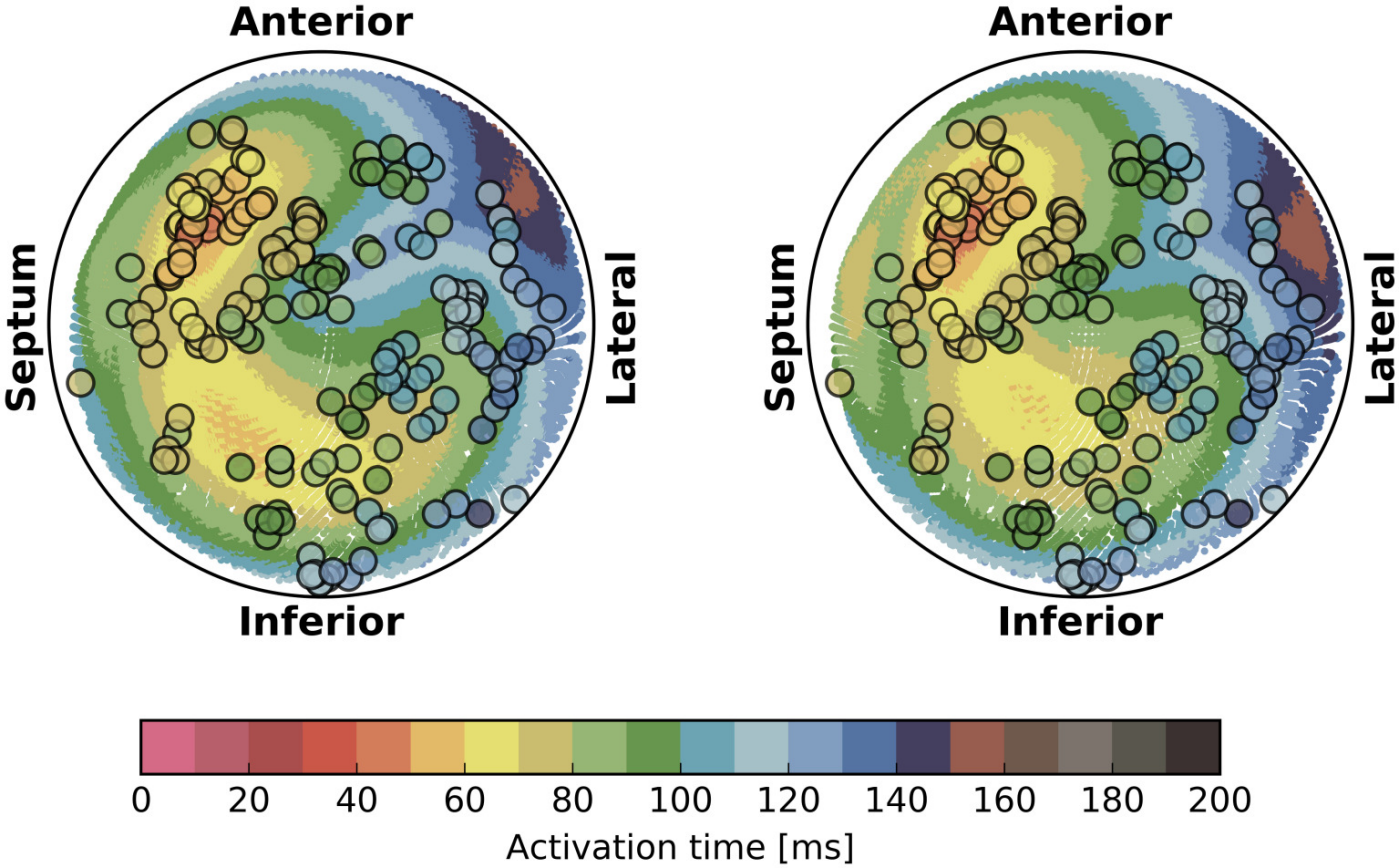
**Bull's eye plot of the activation time at the LV endocardium for the eikonal model (on the left)** and the reaction-diffusion model **(on the right)**, in the case of Patient 1. The filled circles in the plot represent the measured activation time from the electro-anatomical map.

The activation times were compared in terms of maximum absolute error quantiles at the nodes and specific timing markers of cardiological interest at the LV endocardium. In particular, we measured the Trans-Septal Time (TST) and the Total Activation Time (TAT), which are the time of initial and the latest time of activation of the LV endocardium, respectively, and the QRS complex duration (QRSd), defined as the last activation time. Results are reported in Table 4. A bull's eye plot of the LV endocardium is also provided in Figure 7 for an average case in terms of error (Patient 1).

**Table 4:** **Activation times markers for the eikonal model compared to the reaction-diffusion equation (between parentheses), and quantiles of the absolute error between the two models, computed node-wise**.

**Patient**	**QRSd [ms]**	**TST [ms]**	**TAT [ms]**	**Error quantiles [ms]**		
				**0.50**	**0.75**	**0.90**
1	155.48 (157.39)	45.93 (39.10)	109.54 (118.29)	7.11	10.77	12.91
2	184.20 (225.03)	20.15 (19.76)	136.58 (136.66)	5.02	8.95	11.99
3	137.95 (137.92)	42.04 (40.16)	87.98 (88.67)	2.58	5.06	8.06
4	143.15 (145.41)	38.47 (36.85)	104.67 (107.94)	3.76	6.15	9.15
5	144.64 (146.22)	32.01 (31.42)	105.84 (109.17)	2.64	4.52	6.83
6	165.60 (165.58)	30.04 (28.30)	128.40 (128.99)	2.88	5.02	7.72

*The markers are the QRS duration (QRSd), the Trans-Septal Time (TST), which measures the time of first activation of LV endocardium, and Total Activation Time (TAT), which marks the latest activation time in the LV endocardium*.

The overall propagation direction was captured for all the patients. The total activation time differed by 3 to 5 ms, being higher in the reaction-diffusion model. Thus, in the latter the excitation front was slower. The scaling parameter α was roughly 2 cm ms^−1^ mS^−1/2^ in all cases, with a variability within 1 %.

The isochrones were very similar in shape. The absolute error was well-distributed in the anatomy, although we observed a slightly larger error at the apex and in the LV. The EASs regions also differed, with the eikonal solution more ellipsoidal than the R-D model.

On average, we found that 90% of the nodes had an absolute error lower than 10 ms. QRSd, TAT and TST were very similar in all the cases, with the exception of patient 2, where QRSd with the R-D model was very high. The difference was due to the lower resolution geometry employed in the eikonal model (1 vs. 0.2 mm of the R-D model), which may introduce artifacts in the propagation. In patient 2, the anatomy included a thin, late-activated ventricular bundle stemming from the posterior LV free-wall, observed in MRI. In the downsampled geometry, this bundle was poorly reproduced resulting in a shorter activation time.

For the sake of completeness, in Figure 7 we also reported the electro-anatomical map performed on Patient 1 (NOGA® XP, Biologic Delivery Systems, Division of Biosense Webster a Johnson & Johnson Company, USA). Quantitatively, the mismatch between the measured time and the simulated time was on average 11 ± 8 ms for the R-D model and 14 ± 8 ms for the eikonal model. The fitting was performed on the R-D model in previous work (Potse et al., 2014; Nguyên et al., 2015).

The ECGs were computed according to the standard 12-lead ECG definition (Malmivuo and Plonsey, 1995; Macfarlane, 2012), i.e., three limb leads (I, II, III), three “augmented” limb leads (aVR, aVL, aVF) and six precordial leads (*V*_1_ to *V*_6_), with time resolution of 0.5 ms over a time window of 600 ms. The 12 lead fields were computed according to Equation (12) on a 3d finite-difference grid at 1 mm resolution. The total number of voxels was on average 30 million. Each computation took roughly a minute and 80 GB of memory in total using 576 MPI processes on 16 nodes of a Cray XC30 supercomputer.

**Figure 8:**
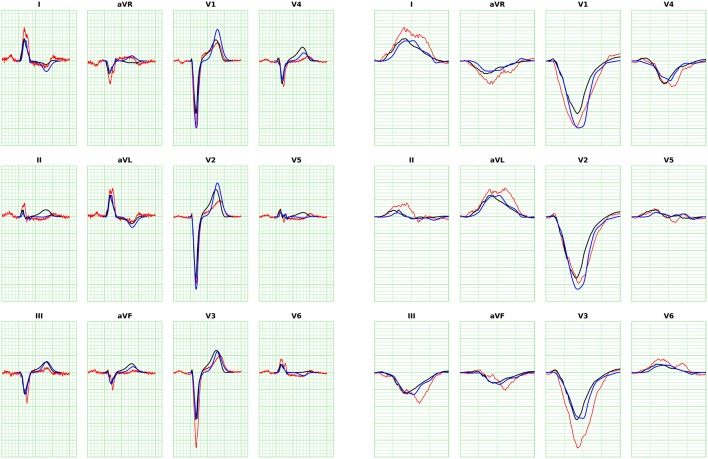
**ECG for Patient 1 (left)** and magnification of QRS complex **(right)**, computed with FM. The blue curve is the solution with the eikonal solver and the lead fields formulation. The black curve is the bidomain solution. The minor grid lines are spaced 40 ms on the horizontal axis and 0.1 mV on the vertical axis. The measured ECG of the patient is shown in red.

The ECGs for all the patients were computed from the activation time provided by the eikonal model and then compared against the bidomain solution. We adopted the action potential template in Equation (20) with uniform parameters in space with values from Table 1, enabling the computation with the FM. The APD was tuned per patient to match the T-wave onset. An example for Patient 1 is provided in Figure 8, where we also report the measured ECG from the patient and a magnification of the QRS complex. In this case the APD was 250 ms.

**Figure 9:**
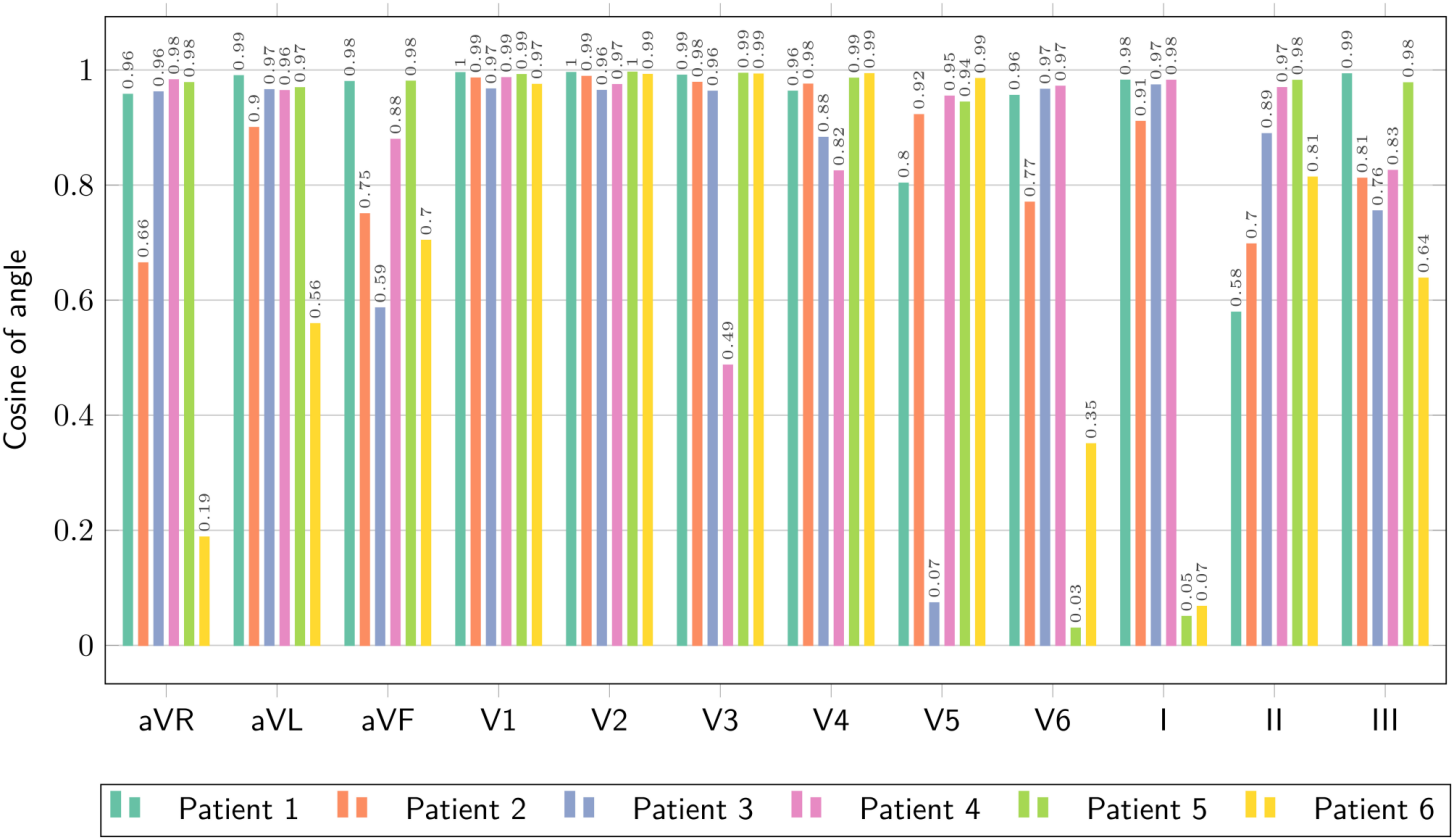
**Comparison of ECG obtained with the bidomain model and with the eikonal equation with lead fields, only limited to the QRS complex, in terms of the normalized dot-product of the two signals, as defined in Equation (25)**.

The error analysis was limited to the QRS complex, since T-waves cannot be properly modeled by the eikonal approach. We compared the ECGs computed with the R-D model and the eikonal model in terms of: difference in maximum amplitude, area and positivity range, correlation, and *L*^2^-dot product of the *L*^2^-normalized ECGs, defined as follows:

(25) $$\frac{\int_{0}^{T}V_{1}(t)V_{2}(t)\text{d}t}{{\left(\int_{0}^{T}V_{1}^{2}(t)\text{d}t\right)}^{1/2}{\left(\int_{0}^{T}V_{2}^{2}(t)\text{d}t\right)}^{1/2}},$$

where *V*_1_(*t*) and *V*_2_(*t*) are two signals. The closer the *L*^2^-dot product to unity, the closer the two signals. We reported the *L*^2^-dot product for all the leads and all the patients in Figure 9.

The ECGs computed with the SM and FM were very similar (not shown), with a small difference in amplitude in the QRS complex (FM was lower). The QRS complex was correctly captured in most of the leads for all the patients except for patient 6, where we observed large deviations in limb leads and moderate differences in precordial leads, whilst the activation map was fairly similar between the two approaches. This patient has a scar in the LV free wall that was modeled in the R-D model as purely passive. We suspect that in the downsampled geometry adopted by the eikonal model the scar was particularly jagged, hence affecting the ECG. In some cases our model provided a slightly larger amplitude in the signal than the bidomain model. Only in one case, for lead V5 of patient 5, we observed discordant QRS complexes. The discrepancies were particularly marked in leads with small amplitude. For patient 2, the discrepancy in the QRS duration was not present when calculated according to the ECG rather then activation map. As reported above, this was due to a ventricular bundle that did not affect the ECG. In general, the correlation between the bidomain model and the proposed model was very good in most of the leads, and the error lower than 0.5 mV.

### 3.4. Performance and scaling

The runtimes of the proposed eikonal solver and ECG solvers on the GPU were tested for six patient-specific geometries with 1-mm resolution.

We tested our code on two different Nvidia GPUs: a low-end (LE) GeForce GT 650M on a laptop (384 cores, 950 MHz clock, 0.73 Tflops of theoretical peak performance), and a high-end (HE) GeForce GTX 1080 (2,560 cores, 1,607 MHz clock, 8.2 Tflops of peak performance) on a local cluster node.

**Figure 10:**
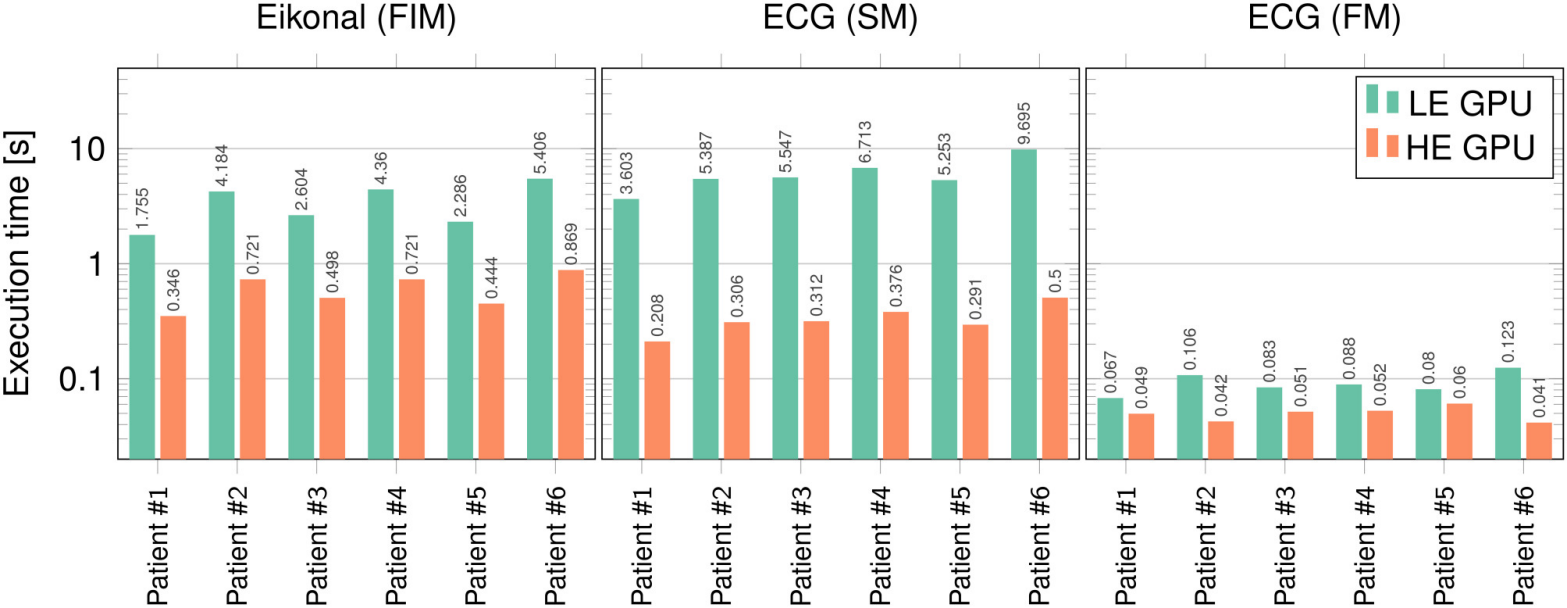
**Runtimes (in seconds) for FIM, SM, and FM on the LE and HE GPU**. Logarithmic scale is used on the vertical axis.

In Figure 10 runtimes of the Fast Iterative Method (FIM) for solving the eikonal model, and the Fast Method (FM) and Simple Method (SM) for the ECG computation are shown, on both the LE and HE GPU. In Table 5 we report the speedup of HE GPU vs. LE GPU for FIM, FM and SM, and the speedup of the FM vs. SM for ECG computation. The reported variability in the computational time was due to the size of the heart and to the number of EASs. This was apparent for patient 6, whose heart was extremely dilated and with 4 EASs reported by the electro-anatomical map.

**Table 5:** **Speedup factors of HE GPU vs. LE GPU for FIM, SM and FM, and FM vs. SM on both HE and LE GPU**.

		**Speedup factor**						
	**Patient**	**1**	**2**	**3**	**4**	**5**	**6**	**Average**
HE vs. LE GPU	FIM	5.1	5.9	5.3	6.1	5.2	6.2	5.6
	SM	17.3	17.6	17.8	17.9	18.1	19.4	18.0
	FM	1.4	2.5	1.6	1.7	1.5	3.0	1.9
FM vs. SM	HE GPU	4.2	7.3	6.1	7.2	4.8	12.2	7.0
	LE GPU	53.8	51.3	67.7	76.3	57.1	79.5	64.3

## 4. Discussion

A critical issue in computational cardiac electrophysiology is the complexity of the reaction-diffusion model. It is nearly impossible to consider this model for inverse problems, unless appropriate model reduction techniques are employed (Gerbeau et al., 2015; Quarteroni et al., 2017). Patient-tailoring of such models (Krueger et al., 2010; Potse et al., 2014) is also a tedious and time-consuming activity. The most expensive part of the procedure is the localization of early activation sites (EASs) and the determination of the local conduction velocity (CV). The proposed combination of an eikonal model for the activation sequence and an ECG computed with lead fields is a viable alternative for such a purpose. Given the EASs and the CV the activation map is found in a few seconds. Since the lead fields do not depend on these parameters, they can be computed once and repeatedly used for ECG simulation. The method can be used as part of inverse models to estimate patient-specific parameters and to make patient-specific predictions of cardiac activation patterns for complex procedures such as cardiac resynchronization therapy (CRT) (Pashaei et al., 2011). The computational power of the current parallel eikonal solver is so high, that our proposed methods are even suitable for real-time interactive simulations (Sermesant et al., 2007; van Oosterom et al., 2011) and therapy simulations based on virtual reality (Pernod et al., 2011).

The proposed method compares adequately to the bidomain model, in terms of accuracy of the delivered solution. The average modeling error in the patient-specific context was roughly 15 % for the activation map (Table 4), without adapting the parameters to compensate the numerical error. The ECG was less accurate in absolute value, but the overall electrocardiographic features of the QRS complex were generally captured. It is well-known that the ECG is very sensitive even to small variations in the activation map. Thus, the mismatch between the reaction-diffusion model and the eikonal model was clearly visible, especially in leads where the potential is small.

The T-wave was almost never captured by our model. This was expected, since our model assumed that the repolarization follows exactly the depolarization, which was not the case in the reaction-diffusion model.

Finally, scalability was excellent for SM, and very good for the FIM and FM. Moreover, the SM and the FM provided very similar QRS complexes for all the patients and with different resolutions, but the FM is two orders of magnitude faster than the SM.

### 4.1. Appropriateness of the eikonal model

The eikonal model can be much faster than a reaction-diffusion equations for three reasons. First, the computational domain is reduced by one dimension, because the dependent variable is no longer a function of time. Second, the mesh can be coarser (1 vs. 0.1 mm), which results in much shorter computation times and much lower memory requirements. Last, it does not require computation of the ionic currents, which takes most of the computational time in the reaction-diffusion solution. Pullan et al. (2002) discussed that because the activation time, unlike the transmembrane potential, does not exhibit internal layers and is a smooth function in the cardiac tissue, it can be assumed that a 1-mm spatial scale is fine enough to accurately reconstruct the changes of the activation time as well as the changes in the speed of the wavefront propagation. They also discussed that sharp changes in the propagation speed can occur, for example when a wavefront collides with another wavefront or boundary, but the fine details of the wavefront shape in these small collision regions are not expected to have much influence on the overall ventricular function.

The numerical comparison on a simple geometry has shown that the eikonal equation is able to approximate the activation times of a bidomain model very accurately, and that there is no advantage in using the full bidomain formulation when modeling the excitation wavefront propagation if the conduction velocities are given.

The disadvantages of the eikonal model are obvious: the effects of ionic currents on conduction, for example partial refractoriness, cannot be simulated at all, simulation of reentrant arrhythmia is very difficult, and there is no calcium transient available for a realistic coupling to a mechanical model. Although several alternative eikonal-like models have been proposed in the literature aiming to improve the accuracy in such situations, such as fibrillation (Herlin and Jacquemet, 2011; Pernod et al., 2011) and high-order curvature effects (Dierckx et al., 2011), in our opinion reaction-diffusion models are necessary to study such phenomena.

### 4.2. Sources of differences between models

We believe that most of the discrepancy in the activation map between the R-D model and the proposed model is explained by the numerical error, and only minimally by the physiological phenomena that the eikonal model neglects.

The bidomain equation requires a mesh resolution that is comparable to the excitation front thickness, which depends on the conductivity in the propagation direction (Pezzuto et al., 2016). In practical situations it is in the order of 0.1 mm. In the cross-fiber direction, however, the conductivity is 10-fold lower, resulting in a steeper wavefront with thickness of 25 μm or lower, which the grid could not resolve. The effect is a significantly slower CV and a higher total activation time. In this situation, we estimated a 20 % numerical error in the relative maximum norm, which is substantial.

The eikonal solver was affected by a 5 % error at 1 mm resolution on a simple geometry. Moreover, the activation time was generally overestimated by the solver, similarly to the slower propagation observed in the reaction-diffusion model when solved with a finite-difference scheme. However, it is hard to tell whether the differences between the reaction-diffusion model and the proposed model are due to numerical errors or modeling errors. Very likely it is a mix of the two.

In our opinion, the eikonal model may have been more accurate than the reaction-diffusion model in the presented experiments. A comparison of the two models in Section 3.2 reported a modeling error of about 6 %. A small modeling error is also reported by others for an idealized LV geometry with physiologically motivated fiber architecture (Colli Franzone et al., 2014).

### 4.3. Scalability issues for the FIM and FM

The FIM ran on the HE-GPU on average only 5.6 times faster than on the LE-GPU. This is about half the ideal scaling. This may be explained by the fact that, at each iteration, only a fraction of the threads are active. In the FIM, only the activation times at the active nodes are evaluated, and hence only a few thousands of threads are running concurrently. A lower number of threads implies a lower number of active CUDA blocks and warps, and this may cause a paucity of warps prepared to hide the latencies of the long-lasting operations. The critical parts are the initial phase of the excitation process when the front starts to propagate from the EASs and the final phase when the last cells are activated. In these two phases only tens or hundreds of threads evaluate the solution and the GPU resources are not fully exploited.

Scalability of the FM was also not optimal. The FM ran on the HE-GPU on average only 4.5 times faster than on the LE-GPU. This means that the performance of the FM is more limited by the internal structure of the kernel than by the number of available GPU cores. The kernel generates one thread per cube in the computational domain. Warp divergence arises because individual threads compute their contribution only when the excitation front intersects the cube. With respect to the scaling, not the warp divergence itself is problematic, but rather the fact that different warps may require different numbers of clock cycles to finish their execution. The higher the number of time instants for which the warp thread evaluates the solution, the higher the warp execution time. Due to unbalanced execution times of different warps in the computational domain the linear scaling of the FM was broken.

## 5. Conclusions

We proposed a combination of an eikonal model for action potential propagation and an ECG simulation method based on lead fields. It can simulate an activation sequence and ECG in about 3 s on a GPGPU desktop platform (less than a second on HE-GPGPU). This method is suitable as a component of an inverse electrocardiographic model in an HPC context, but may in the near future also become practical within interactive ECG simulation tools. While we based our work largely on classical ideas, we believe that the following aspects are novel.

- We proposed a new method to compute the ECG, based on the marching cubes method, which allows for several orders of magnitude speedup.
- We compared the results of the eikonal model to those of a monodomain reaction-diffusion model, pointing out the difficulties of such a comparison.
- We compared the simulated ECGs with those of a bidomain torso model and identified the most important causes of differences.
- We discussed how the proposed methods can be implemented on a GPGPU.
- We showed that the proposed methods can simulate a highly realistic ECG in a few seconds.

## Author contributions

The ideas behind this work were initially proposed by MP, who also provided the numerical experiments with propag-5 and the patient's anatomies. SP designed the numerical method, drafted the implementation and performed the simulations. PK implemented the GPGPU code and optimized it. He wrote the initial version of the manuscript, then extended and revised by SP and MP to the present state. FP contributed on several occasions to the manuscript by reviewing it. AA and RK respectively reviewed the clinical outcome and numerical aspects of the manuscript.

## Funding

This work was supported by grants from the Swiss National Supercomputing Centre (CSCS) under project IDs s397, s598, and s669. PK was supported by the Sciex-NMS fellowship “CATION,” project code 14.158. The authors gratefully acknowledge financial support by the Theo Rossi di Montelera Foundation, the Metis Foundation Sergio Mantegazza, the Fidinam Foundation, and the Horten Foundation to the Center for Computational Medicine in Cardiology. The authors also thank the Swiss PASC initiative (Platform for Advanced Scientific Computing) for their support. This work was also partially supported by a restricted grant of Biologic Delivery Systems, Division of Biosense Webster a Johnson & Johnson Company.

### Conflict of interest statement

FP has received research grants from Medtronic, St. Jude Medical, Sorin, MSD, and Biotronik. AA has been a consultant to Medtronic, Boston Scientific, LivaNova and St. Jude, and has received speakers' fees from Medtronic, Boston Scientific and LivaNova. The other authors declare that the research was conducted in the absence of any commercial or financial relationships that could be construed as a potential conflict of interest.
