# Supplementary material for: Evaluation of a Rapid Anisotropic Model for ECG Simulation
Source: Front Physiol. 2017 May 2;8:265. doi: 10.3389/fphys.2017.00265 (PMC5411438; doi:10.3389/fphys.2017.00265)
Supplement: Supplementary file 1 [file Presentation1.pdf]

Evaluation of a rapid anisotropic model for ECG simulation: supplementary material

Simone Pezzuto Peter Kaľavský Mark Potse Frits W. Prinzen
Angelo Auricchio Rolf Krause

Explicit formula for ECG in convergence test

The function $w(t)$ defined in (14) and used in the computation of the ECG in equation (15) can be computed explicitly in the case considered in the first experiment of section 3.1. To see this, we first observe that the boundary of the activated region $\mathcal{V}_t := \{x \in \Omega \colon \Psi(x) < t\}$ is subdivided into activation front $\Psi^{-1}(t)$ and the activated boundary $\mathcal{B}_t := \mathcal{V}_t \cap \partial\Omega$, thus

$$w(t) := -\int\limits_{\Psi^{-1}(\xi)} \mathbf{G}_\mathrm{i}\nabla Z \cdot \mathbf{n}\,\mathrm{d}\Sigma = -\int\limits_{\Psi^{-1}(\xi)\cup\mathcal{B}_t} \mathbf{G}_\mathrm{i}\nabla Z \cdot \mathbf{n}\,\mathrm{d}\Sigma + \int\limits_{\mathcal{B}_t} \mathbf{G}_\mathrm{i}\nabla Z \cdot \mathbf{n}\,\mathrm{d}\Sigma. \tag{1}$$

Since $\Psi^{-1}(\xi) \cup \mathcal{B}_t$ equals $\partial\mathcal{V}_t$, we can apply the divergence theorem to the first term

$$w(t) = \int_{\mathcal{V}_t} \nabla \cdot (\mathbf{G}_\mathrm{i}\nabla Z)\,\mathrm{d}x + \int_{\mathcal{B}_t} \mathbf{G}_\mathrm{i}\nabla Z \cdot \mathbf{n}\,\mathrm{d}\Sigma. \tag{2}$$

The volumetric term cancels in our case, since $\mathbf{G}_\mathrm{i}\nabla Z$ is constant. We remark that equal anisotropy ratio would be sufficient to cancel this term in general [1]. The boundary term simplifies also, since our domain is a cube and all the faces $F_i^\pm$ are planar with constant normal direction and oriented along the axes $\mathbf{e}_i$:

$$w(t) = (\mathbf{G}_\mathrm{i}\nabla Z) \cdot \sum_{i=1,2,3} \mathbf{e}_i \Big(|\mathcal{B}_t \cap F_i^+| - |\mathcal{B}_t \cap F_i^-| \Big). \tag{3}$$

In the numerical experiment $\nabla Z = -\mathbf{e}_1$, $\mathbf{G}_\mathrm{i} = \sigma_\mathrm{it}\mathbf{I} + (\sigma_\mathrm{il} - \sigma_\mathrm{it})\mathbf{e}_3 \otimes \mathbf{e}_3$ thus $w(t)$ is proportional to the difference between the activated area on the bottom face and the activated area on the top face:

$$w(t) = \sigma_\mathrm{it}\Big(\big|\{\Psi(0,y,z) < t\}\big| - \big|\{\Psi(\ell_\mathrm{x},y,z) < t\}\big| \Big), \qquad (y,z) \in [0,\ell_\mathrm{y}] \times [0,\ell_\mathrm{z}]. \tag{4}$$

The activated area is simple to compute when it is an axis-aligned ellipse centered in the face, e.g. when the activation map is as follows:

$$\Psi(x,y,z) = \sqrt{\frac{(x-x_\mathrm{c})^2}{s_\mathrm{x}^2} + \frac{(y-\ell_\mathrm{y}/2)^2}{s_\mathrm{y}^2} + \frac{(z-\ell_\mathrm{z}/2)^2}{s_\mathrm{z}^2}}. \tag{5}$$

In the numerical experiment we have $s_\mathrm{x} = s_\mathrm{y} = \alpha\sqrt{\sigma_\mathrm{t}/\beta}$ and $s_\mathrm{z} = \alpha\sqrt{\sigma_\mathrm{l}/\beta}$. After tedious but straightforward computation the final form of exact solution reads as follows:

$$w(t) = \sigma_\mathrm{it}\ell_\mathrm{y}\ell_\mathrm{z}\Big(f\big(t, x_\mathrm{c}/s_\mathrm{x}\big) - f\big(t, (\ell_\mathrm{x} - x_\mathrm{c})/s_\mathrm{x}\big) \Big) \tag{6}$$

where we defined the following auxiliary functions:

$$g(c,r) = \begin{cases} 4 & \text{if } r \geq \sqrt{1+1/c^2} \\ c\pi r^2 & \text{if } (c \leq 1 \wedge r \leq 1) \vee (c > 1 \wedge cr \leq 1), \\ 2c\big(u + r^2 \operatorname{arccsc} r\big) & \text{if } r > 1 \wedge cr \leq 1, \\ \frac{2v}{c} + cr^2\big(\pi - 2\arctan v\big) & \text{if } cr > 1 \wedge r \leq 1, \\ \frac{2}{c}\big(c^2u + v + c^2r^2(\operatorname{arccsc} r - \arctan v)\big) & \text{otherwise,} \end{cases} \tag{7}$$

$$f(t,s) = \begin{cases} g\big(c, 2s_\mathrm{y}/\ell_\mathrm{y}\sqrt{t^2 - s^2}\big) & \text{if } t > 0 \wedge t^2 > s^2, \\ 0 & \text{otherwise,} \end{cases} \tag{8}$$

with $u = \sqrt{r^2-1}$, $v = \sqrt{c^2r^2-1}$ and $c = \frac{\ell_\mathrm{y} s_\mathrm{z}}{\ell_\mathrm{z} s_\mathrm{y}}$. In particular, $g(c,r)$ is the area of the intersection between the ellipse of semi-axes 1 and c and the square $[-1,1]^2$.

References

[1] Colli Franzone, P., Pavarino, L. F., and Scacchi, S. *Mathematical Cardiac Electrophysiology.* Springer, 2014.
